# Case report: A sustained survival benefit of third-line immunotherapy for refractory thymic carcinoma

**DOI:** 10.3389/fonc.2024.1326006

**Published:** 2024-07-23

**Authors:** Mi Meng, Bo Yu, Jie Luo, Yuju Bai, Lin Li, Shicheng Chen, Sisi He, Hu Ma

**Affiliations:** ^1^ Department of Thoracic Oncology, The second Affiliated Hospital of Zunyi Medical University, Zunyi, China; ^2^ Department of Urology, The second Affiliated Hospital of Zunyi Medical University, Zunyi, China; ^3^ Department of Radiation Oncology, Guiqian International General Hospital, Guiyang, China

**Keywords:** sintilimab, thymic carcinoma(TC), therapeutic option, case report, anti-PD-1

## Abstract

Thymic carcinoma (TC) is an uncommon type of thymic epithelial tumors. Patients with relapsed or refractory TCs have a poor prognosis. Immune checkpoint inhibitor monotherapy can be applied as a second-line treatment for such cases. This study reported a TC patient who did not respond to conventional chemotherapy and radiotherapy but achieved prolonged partial remission lasting 17 months following the third-line treatment with anti-programmed cell death-1 inhibitor sintilimab. This patient did not experience any serious side effects associated with sintilimab treatment. The above results demonstrated that sintilimab could be a feasible therapeutic option for refractory TC patients.

## Introduction

Thymic carcinoma (TC) is a rare subgroup of aggressive thymic epithelial tumors according to the World Health Organization (WHO) 2015 classification (1). It is highly prone to distant metastases, with a five-year survival rate for stage IV patients of 21.2% (2). The standard treatment for metastatic or recurrent TC is systemic chemotherapy, and paclitaxel plus platinum is the preferred first-line regimen, with an objective response rate (ORR) ranging from 22% to 36% (3). Nevertheless, there is no standard second-line therapy. Previous studies showed that gemcitabine plus capecitabine and pemetrexed had a response rate of 19% to 36% for small sample researches with refractory TC (4, 5). Similarly, lenvatinib and sunitinib showed limited efficacy (6, 7). Therefore, a novel treatment strategy is urgently needed for refractory TC.

The PD-L1 expression level could predict immune efficacy. Pembrolizumab can effectively treat TC, with a median progression-free survival (PFS) time of 3.8 to 6.1 months (8), indicating that immunotherapy might be used as adjuvant therapy for refractory TC. Moreover, the application of immunotherapy in thymic epithelial tumors (TETs) has been quoted in National Comprehensive Cancer Network (NCCN) guidelines (8–11).

Sintilimab (Innovent Biologics, Suzhou, China) is a humanized monoclonal antibody that blocks the interaction between programmed cell death-1 (PD-1) and programmed death ligand 1 (PD-L1). It has been approved in China for the treatment of non-small cell lung cancer (NSCLC), hepatocellular carcinoma (HCC), Hodgkin’s lymphoma, and various solid tumors (12).

We reported a female patient who suffered from refractory TC and showed no response to chemotherapy and radiotherapy (RT). She received sintilimab monotherapy and achieved sustained partial remission (PR) lasting 17 months. Moreover, the patient had almost no sintilimab-related adverse events.

## Case presentation

**Figure 1:**
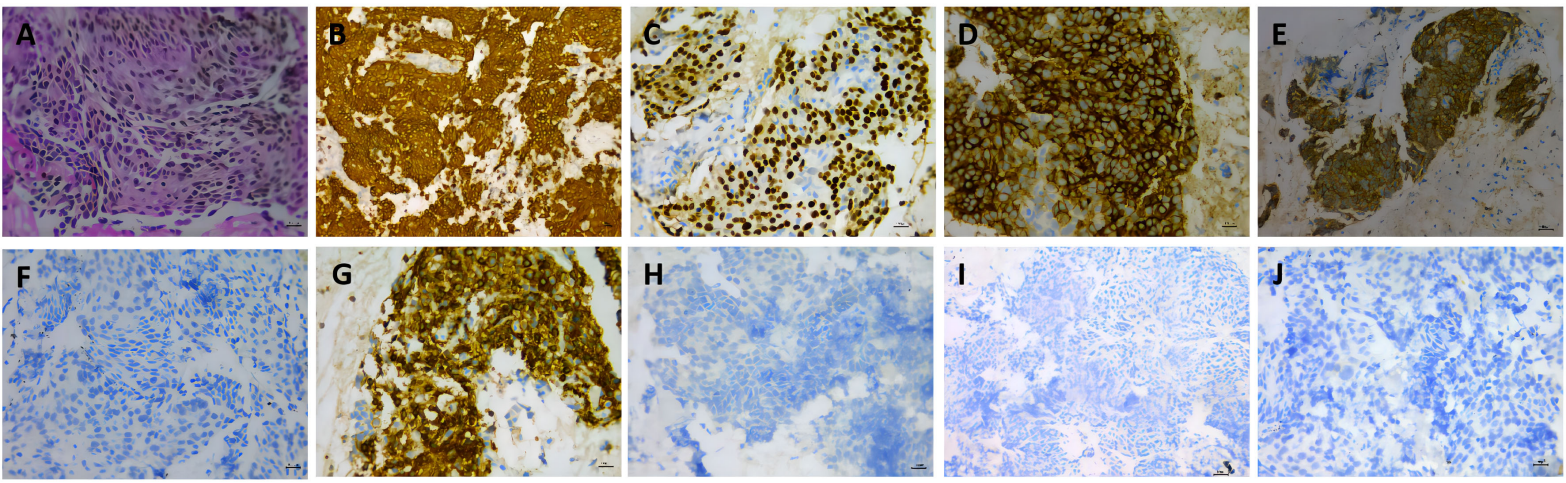
Histopathologic stains from thymic **(A–J)**. **(A)** hematoxylin and eosin(400X); **(B)** showed the thymic tumor cells were cytoplasmic positive for CD5/6 (200X); **(C)** showed the thymic tumor cells were cytoplasmic positive for P63 (400X); **(D)** showed the thymic tumor cells were cytoplasmic positive for CD117 (400X); **(E)** showed the thymic tumor cells were cytoplasmic positive for CD5(400X); **(F)** showed the thymic tumor cells were cytoplasmic negative TTF1(400X); **(G)** showed the thymic tumor cells were cytoplasmic positive forLCA(400X); **(H)** showed the thymic tumor cells were cytoplasmic negative TdT(400X); **(I)** showed the thymic tumor cells were cytoplasmic negative NapsinA(200X); **(J)** showed the thymic tumor cells were cytoplasmic negative CK7 (400X).

**Figure 2:**
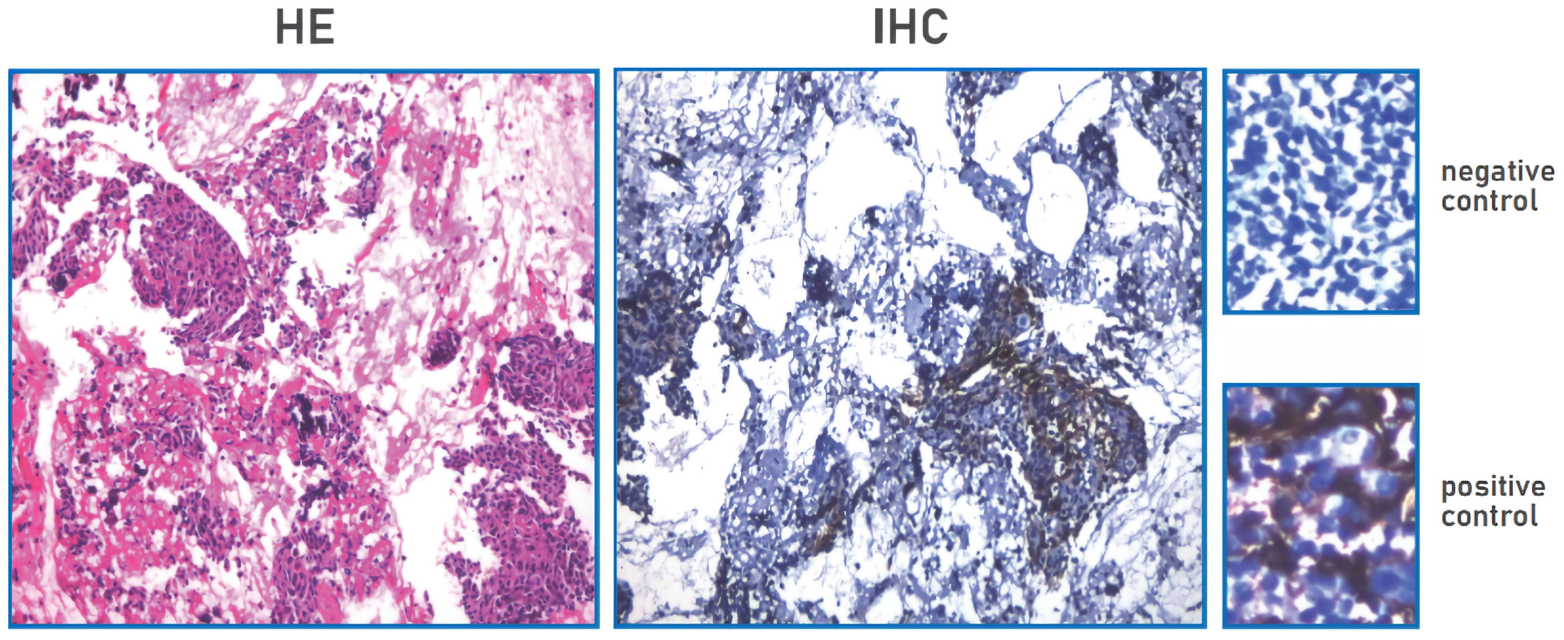
PD-L1 expression TPS negative, TPS<1% (TPS<1%); PD-L1 expression CPS positive, CPS≥1(CPS=1).

A 53-year-old female was admitted to our hospital in May 2019 due to shortness of breath for two months. The contrast-enhanced chest computed tomography (CT) revealed a large space-occupying lesion involving the heart and large vessels in the anterior mediastinal region. Histopathological stains of the thymic biopsy specimen revealed CK5/6 (+++), P63 (+++), CD117 (+++), CD5 (+++), NapsinA (–), CK7 (-), TTF1 (-), Vimentin (-), LCA (+++), and TdT (-) (**Figure 1**), indicating squamous cell carcinoma. The PD-L1 expression level was that the tumor proportion score (TPS) was found to be less than 1%, and the combine positive score (CPS) was found to be 1% (**Figure 2**). Therefore, the patient was diagnosed with Masaoka stage IIIB thymic squamous cell carcinoma. The neoadjuvant chemotherapy regimen of carboplatin AUC 6 plus paclitaxel 175 mg/m^2^ was administered every three weeks to reduce tumor size and make it resectable. The response evaluation criteria in solid tumors (RECIST) evaluated the treatment effect as stable disease (SD) after three treatment cycles. However, the patient could not tolerate the chemotherapy, and the neoadjuvant treatment was suspended.

**Figure 3:**
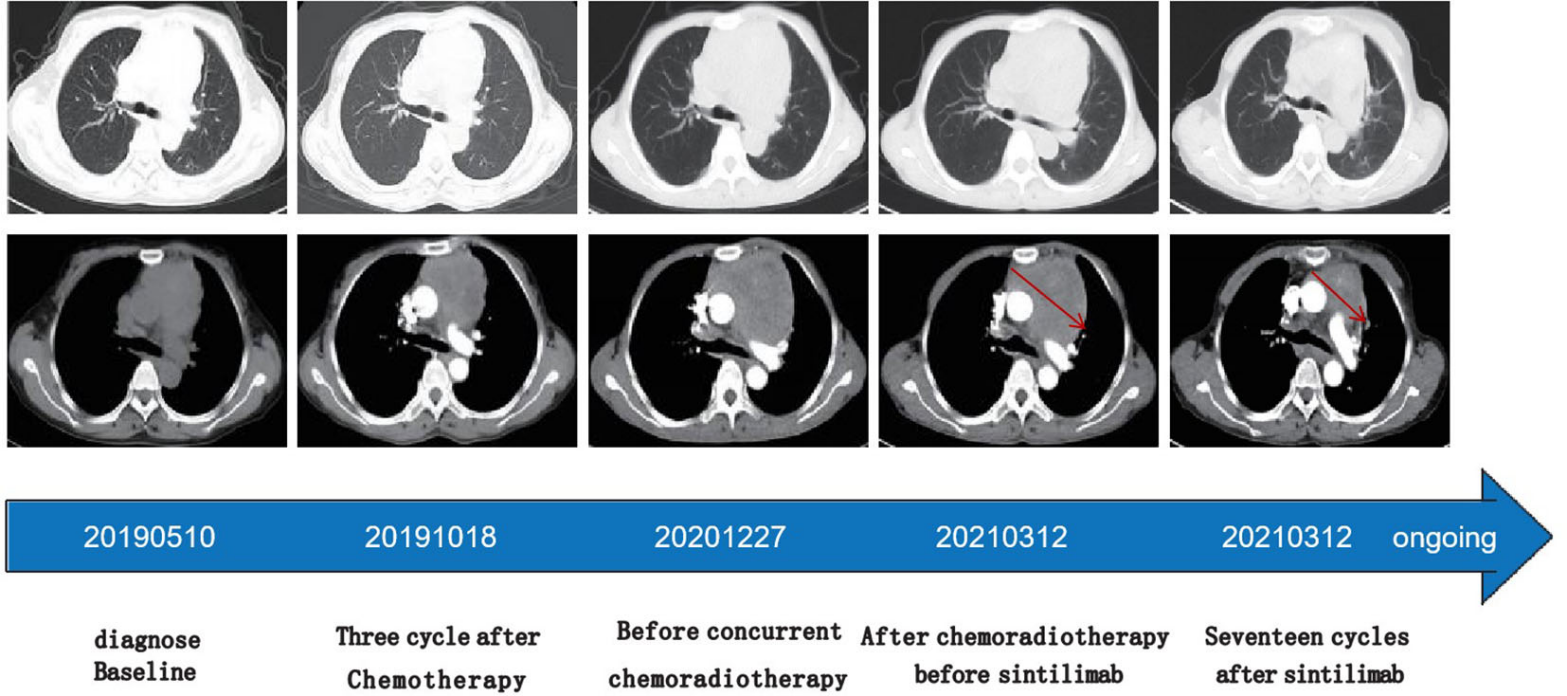
Time line of treatments of the patient and changes in CT scan of thymic carcinoma during treatments Chest computed tomography (CT) revealed a clearly reduced the size of anterior mediastinum lesion indic- ated by red arrows, with a maximum diameter decreasing from 99 mm to 52 mm.

**Figure 4:**
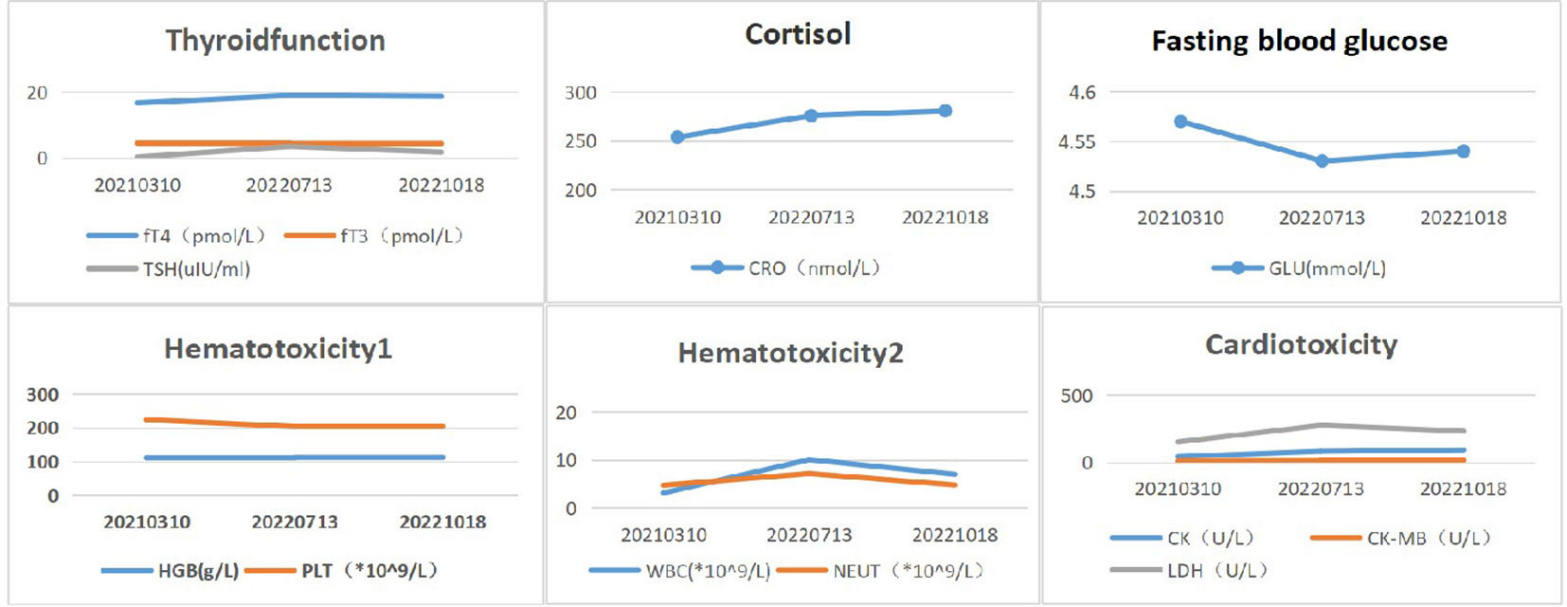
Biochemical and hematological changes after immunotherapy. Reference range:fT4 (8.7-17.3 pmol/L), fT3(2.8-7.1pmol/L), TSH(0.27-4.2uIU/ml), CRO(172-497nmol/L), GLU(3.9- 6.1mmol/L), HGB(115-15 0g/L), PLT(100-300*10^9/L), WBC(3.5-9.5*10^9/L), NEUT(1.8-6.3*10^9/ L), CK(26-140U/ L), CK-MB (0-24 U/L), LDH(140-271U/L).

The patient’s shortness of breath was significantly worse in December 2020, with a larger anterior mediastinum lesion. Therefore, the case received concurrent chemoradiotherapy with cisplatin (60 mg/m^2^ on day 1) plus etoposide (120 mg/m^2^ on days 1-3) plus intensity-modulated radiation therapy (300 Gy each time, 20 times). However, the anterior mediastinum lesion did not significantly shrink, and the patient was deemed to have refractory TC. The patient’s shortness of breath was significantly relieved in April 2021 (3 weeks after initial sintilimab), following administration of 200mg of sintilimab every three weeks. Chest computed tomography (CT) revealed a clearly reduced the size of anterior mediastinum lesion, with a maximum diameter decreasing from 99 mm to 52 mm (**Figure 3**). Therefore, the treatment response was classified as partial response (PR). No severe side effects appeared during immunotherapy for 17 months (**Figure 4**).

## Discussion

This patient presented a partial remission for 17 months after receiving 200 mg of third-line sintilimab every three weeks. Sintilimab was not recommended for metastatic or recurrent TC. Our treatment plan was based on the patient’s resistance to chemoradiotherapy, and that sintilumab was the standard treatment for metastatic or recurrent lung squamous cell carcinoma. Moreover, the NCCN guidelines have recommended the anti-PD-1 inhibitor pembrolizumab monotherapy as the second-line treatment of TC. Besides, patients could afford the long-term use of sintilimab due to its economic advantages. This patient did not develop any significant side effects from radiation, chemotherapy, or immunotherapy.

The patient had a good prognosis after receiving sintilimab as the third-line treatment. Platinum-based chemotherapy is the standard treatment for advanced, unresectable TC, however, it has a short effect (13). Additionally, the treatment options are limited after the failure of platinum-based chemotherapy. Immune checkpoint inhibitors (ICIs) have recently shown promising efficacy in many solid tumors and have become the standard treatment for many cancers. High PD-L1 expression (36-80%) has been reported in TC cases (14–17), suggesting that the anti-PD-1/PD-L1 inhibitors might effectively treat unresectable TC. Despite having one of the lowest mutation rates among cancers (18), TETs had high PD-L1 levels, which could provide strong evidence for the ICI application (19). PD-L1 positivity (≥ 25% of tumor membrane expression) was frequently found in TETs, and a higher PD-L1 level was associated with longer overall survival rates (20). However, our patient had a PD-L1 level of less than 1%, suggesting that immunotherapy was not just beneficial for metastatic TC patients with PD-L1 positivity. There may be several reasons for this case. On the one hand,PD-L1 expression is not an absolute indicator of immune efficacy. PD-L1 expression exhibits intratumoral heterogeneity, that is to say, expression of PD-L1 may vary in slices of the same tissue. Moreover, the expression of PD-L1 also undergoes dynamic changes with treatment. Therefore, single point and single sampling may not fully evaluate the PD-L1 expression of tumors. On the other hand, it is widely recognized that some biomarkers can be used independently to predict immunotherapy effect of solid tumor. For instance, MSIH and dMMR are considered as biomarkers of immune response in colorectal cancer (21). TMB is regarded as an immune predictor in TC (22). In many other solid tumors, such as ovary, lung, breast cancer and malignant melanoma,CD8^+^T-TIL,and CD8+T_RM_ cells can be consider as independent predictors of immunotherapy effect (23–26). Nevertheless, more evidences are required to support such a view. Unfortunately, our patient did not undergo genetic testing to understand the relevant mutations, nor did we undergo circulating immune marker testing to predict the effectiveness of immunotherapy. So no genetic information of this patient was available. Therefore, it is unknown whether our patient has genomic alterations which are responded to immunotherapy.

Radiotherapy(RT) has been found to strengthen systemic antitumor responses to immunotherapy, including activating cytotoxic T cells, triggering immunogenic cell death, and enhancing antigen presentation (27). Besides, it could increase PD-L1 expressions and promote effector T cells infiltration (28). Therefore, some cancer patients following RT manifested significant immunogenic patterns (29). This might explain why this case had a poor response to RT alone but responded well to the combination of RT plus immunotherapy. This may also be the reason why the patient in this case, despite having low PDL1 expression, has a good therapeutic effect with PD-L1. Sintilimab is an anti-PD-1 inhibitor. Previous research showed that RT plus immunotherapy yielded a better treatment effect in NSCLC patients (30). Prior case reports showed that sintilimab combined with radiotherapy could effectively treat HCC patients (31). However, there is no data on the treatment effect of immunotherapy after radiotherapy for TC.

Immunotherapy is a promising treatment in refractory TC patients. However, several studies have revealed that immune-related adverse events (irAE) were found in 15% to 62% of TET patients, such as those with myasthenia gravis (3-14%), hypothyroidism (13%), and myocarditis (5%) (8, 9, 11). Our patient received third-line sintilimab with a median PFS of 17 months and no significant irAEs.

The combined or sequential therapy of anti-PD-1 and RT might be a treatment option in refractory TC. Nevertheless, the finding should be evaluated in dedicated prospective studies.

## Patient perspective

The shortness of breath had already affected my daily life and work, causing me great anxiety. This treatment successfully addressed my difficulties; the doctor explanation and professional treatment group during my hospitalization helped me to largely relieved my fear of carcinoma. I was very relieved that the Immunotherapy process went smoothly without complications, and I consider that I received a very successful treatment. I would like to share my medical history and I have signed an informed consent form.

## Conclusion

The study first reported that third-line sintilimab monotherapy could achieve effective disease control and sustained survival benefit in a TC patient, offering a treatment choice for unresectable TC patients with low PD-L1 expressions. Nevertheless, more clinical trials are required to clarify the safety and effectiveness of sintilimab immunotherapy in TC.

## Data availability statement

The original contributions presented in the study are included in the article/supplementary material. Further inquiries can be directed to the corresponding authors.

## Ethics statement

Written informed consent was obtained from the individual(s) for the publication of any potentially identifiable images or data included in this article.

## Author contributions

MM: Writing – original draft, Writing – review & editing. BY: Writing – original draft, Writing – review & editing. JL: Writing – review & editing. YB: Writing – review & editing. LL: Writing – review & editing. SC: Writing – review & editing. SH: Writing – review & editing. HM: Writing – review & editing.
