# Corrigendum: Case report: A sustained survival benefit of third-line immunotherapy for refractory thymic carcinoma

**DOI:** 10.3389/fonc.2024.1516024

**Published:** 2024-12-02

**Authors:** Mi Meng, Bo Yu, Jie Luo, Yuju Bai, Lin Li, Shicheng Chen, Sisi He, Hu Ma

**Affiliations:** ^1^ Department of Thoracic Oncology, The second Affiliated Hospital of Zunyi Medical University, Zunyi, China; ^2^ Department of Urology, The second Affiliated Hospital of Zunyi Medical University, Zunyi, China; ^3^ Department of Radiation Oncology, Guiqian International General Hospital, Guiyang, China

**Keywords:** sintilimab, thymic carcinoma(TC), therapeutic option, case report, anti-PD-1

In the published article, there was an error in affiliation 1. Instead of “Department of Thoracic Oncology, Affiliated Hospital of Zunyi Medical College, Zunyi, China”, it should be “Department of Thoracic Oncology, The second Affiliated Hospital of Zunyi Medical University, Zunyi, China”.

The authors apologize for this error and state that this does not change the scientific conclusions of the article in any way. The original article has been updated.

